# Exploring a New Entity of Single-Agent Pembrolizumab-Associated Hypophysitis

**DOI:** 10.7759/cureus.27763

**Published:** 2022-08-07

**Authors:** Eric Balti, Sarah Verhaeghe, Vibeke Kruse, Stijn Roels, Peter Coremans

**Affiliations:** 1 Department of Endocrinology and Diabetes, VITAZ Hospital, Sint-Niklaas, BEL; 2 Department of Medical Oncology, VITAZ Hospital, Sint-Niklaas, BEL; 3 Department of Molecular Imaging, Pathology, Radiotherapy and Oncology (MIPRO), University of Antwerp, Wilrijk, BEL; 4 Department of Oncology, Antwerp University Hospital, Edegem, BEL; 5 Department of Radiology, VITAZ Hospital, Sint-Niklaas, BEL

**Keywords:** gonadotropin insufficiency, thyrotropic failure, corticotropin deficiency, anterior pituitary failure, auto-immune hypophysitis, pembrolizumab, immune checkpoint inhibitors

## Abstract

Hypophysitis is the inflammation of the pituitary gland primary or secondary to local or systemic disease. It tends to occur more with cytotoxic T-lymphocyte-associated protein 4 inhibitors (10-15% of cases), which is a different entity compared to that associated with anti-program death 1 (anti-PD1) inhibitors. We describe a case of pembrolizumab-associated hypophysitis and conduct a systematic review of the literature.

A 55-year-old woman with stage pT3aN1a (TNM stadium IIIb) melanoma presented with headache, nausea and fatigue three and a half months after starting pembrolizumab. Blood analyses revealed secondary adrenal failure, thyrotropic insufficiency and defective gonadotrophin secretion. An imaging study showed an enlarged pituitary gland with a homogeneous enhancement of the gland and pituitary stalk. Interruption of anti-PD1 therapy and administration of hormonal supplementation lead to clinical, biological and radiologic improvement after eight months.

We identified 17 studies (20 patients) on single-agent pembrolizumab-associated hypophysitis. Patients were treated for melanoma (n=7; 33.3%), urogenital (n=5 ; 23.8%), lung (n=4 ; 19.0%), larynx (n=1 ; 4.8%), pharynx (n=1, 4.8%), breast (n=1, 4.8%) and colon (n=1, 4.8%) neoplasia. The time to onset of pituitary insufficiency was most frequently six months (range 1.5-39.0 months) after treatment initiation. The most prevalent hormonal defect was isolated adrenocorticotropic hormone (ACTH) deficiency. Four cases were reported with multiple central hormonal defects. In those patients, an enlarged pituitary gland was also observed.

Our case has distinct features, including early disease onset after single-agent pembrolizumab initiation, panhypopituitarism and increased pituitary mass. These findings are in contrast with the majority of other cases of pembrolizumab-induced hypophysitis, as most patients present an isolated ACTH deficiency. Whether or not this is a new clinical entity warrants further investigation.

## Introduction

Pembrolizumab is an anti-programmed cell death protein 1-specific (anti-PD-1) monoclonal antibody used in monotherapy or combination therapy for several types of malignancies. Immune-related adverse events may occur in patients treated with anti-PD-1 monoclonal antibodies due to immune system activation. However, hypophysitis is a rarely reported adverse effect of anti-PD-1 monotherapy. Early recognition and treatment of immune-induced hypophysitis are important to prevent life-threatening complications mostly due to secondary adrenal failure [1-3]. In line with this foreword, we describe the clinical course of single-agent pembrolizumab-induced hypophysitis in a 55-year-old woman treated for malignant melanoma. Distinct clinical features were observed, including early onset after starting pembrolizumab, deficiency in three pituitary axes (adrenocorticotropic, thyrotropic and gonadotrophic axis) and increased pituitary mass.

## Case presentation

A 55-year-old woman presented with headache, nausea and fatigue ongoing for two weeks. Seven months prior to the current episode, she was diagnosed with a stage pT3aN1a (TNM stadium IIIb) malignant melanoma of the right groin. Molecular testing did not find a BRAF or NRAS mutation. Surgical excision and removal of the sentinel node were performed. Adjuvant single-agent therapy with pembrolizumab 2 mg/kg every three weeks for one year was subsequently initiated. After just five cycles of immunotherapy, she presented with the symptoms mentioned above. She did not report increased thirst, polyuria or nocturia. On examination, systemic blood pressure was 155/79 mmHg, heart rate was 72 beats per minute, oxygen saturation was 98% on room air and temperature was 35.5°C. Further physical examination was unremarkable.

**Figure 1:**
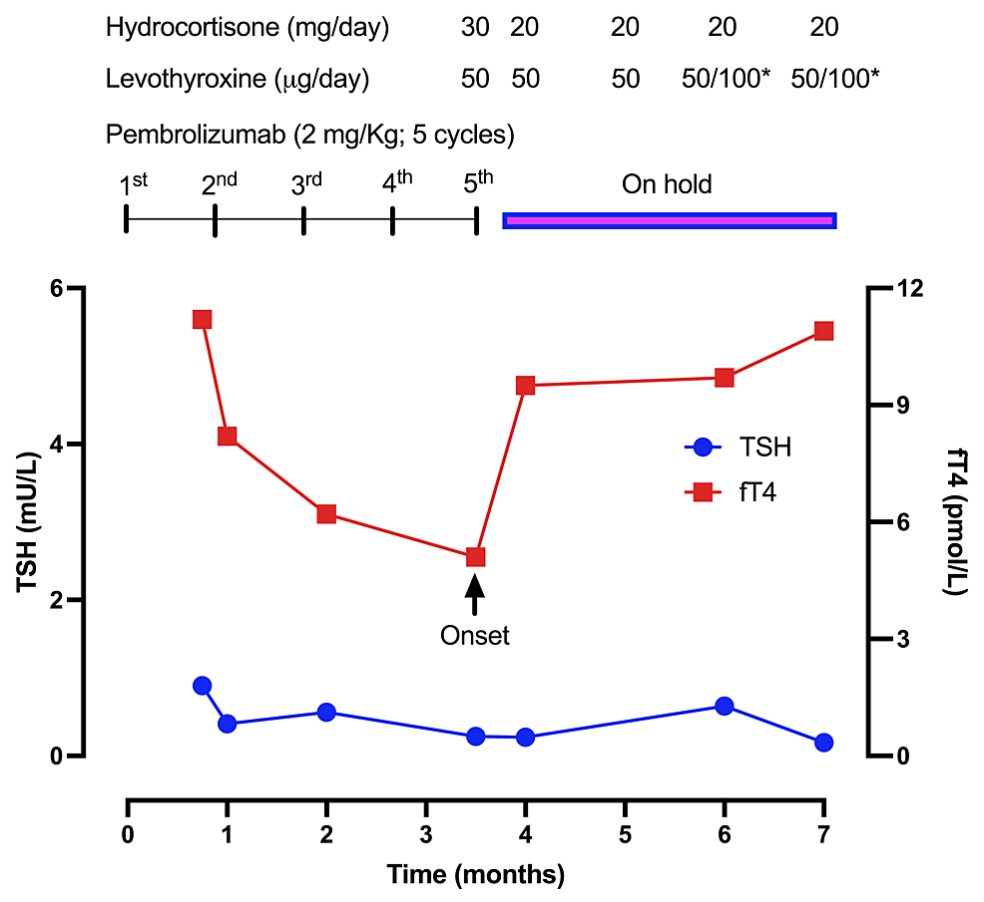
Time-dependent variation of thyroid stimulating hormone (blue line) and peripheral free tetraiodothyronine (red line) from the start of treatment with pembrolizumab (T0) The arrow indicates the time of onset of pituitary failure, including secondary hypothyroidism. * Levothyroxine 50 µg/day 5/7 days and 100 µg 2/7 days

Initial laboratory findings are shown in Table 1. ACTH deficiency was observed as well as secondary hypothyroidism and hypogonadotropic hypogonadism. Very low levels of morning cortisol and ACTH at 0.5 mg/dL (normal range: 6.0 to 30.0 mg/dL) and <5.0 pg/mL (normal range: 10 to 60 pg/mL), respectively, were substantiated on blood analysis. TSH and peripheral thyroid hormone (free T4 and T3) levels also declined after pembrolizumab initiation (Figure 1). Analysis of the gonadotropic axis showed LH lower than 0.2 U/L (normal range: 1.1 to 52.4 U/L) and low FSH at 0.97 U/L (normal range: 5.9 to 72.8 U/L). Growth hormone and IGF-1 levels were normal (Table 1). Likewise, the sodium level was normal. The absence of typical clinical symptoms argued against altered posterior pituitary function. Anti-thyroid peroxidase antibodies were negative.

**Table 1:** Biological characteristics of the reported case at the time of onset of pembrolizumab-induced auto-immune hypophysitis ACTH: adrenocorticotropic hormone, TSH: thyroid stimulating hormone, fT4: free tetraiodothyronine, fT3: free triiodothyronine, LH: luteinizing hormone, FSH: follicular stimulating hormone, CRP: C-reactive protein, IGF-1: insulin-like growth factor 1

Hormone	Patient’s value	Normal range
Morning ACTH	< 5.0 pg/mL	8:00 am: 10-60 pg/mL
Morning cortisol	0.5 mg/dL	8:00 am: 6.0-30.0 mg/dL
TSH	0.25 mU/L	0.35-4.50 mU/L
fT4	5.1 pmol/L	9.3-23.2 pmol/L
fT3	3.72 pmol/L	3.30-6.10 pmol/L
LH	< 0.2 U/L	1.1-52.4 U/L
FSH	0.97 U/L	5.9 -72.8 U/L
Oestradiol	< 11.0 ng/L	11-462.1 ng/L
CRP	16.1 mg/L	< 3.0 mg/L
IGF-1	88.6 ng/ml	44.7-210.0 ng/ml
Growth hormone	1.78 mg/dL	< 8.0 mg/dL
Sodium	138 mmol/L	135-145 mmol/L

**Figure 2:**
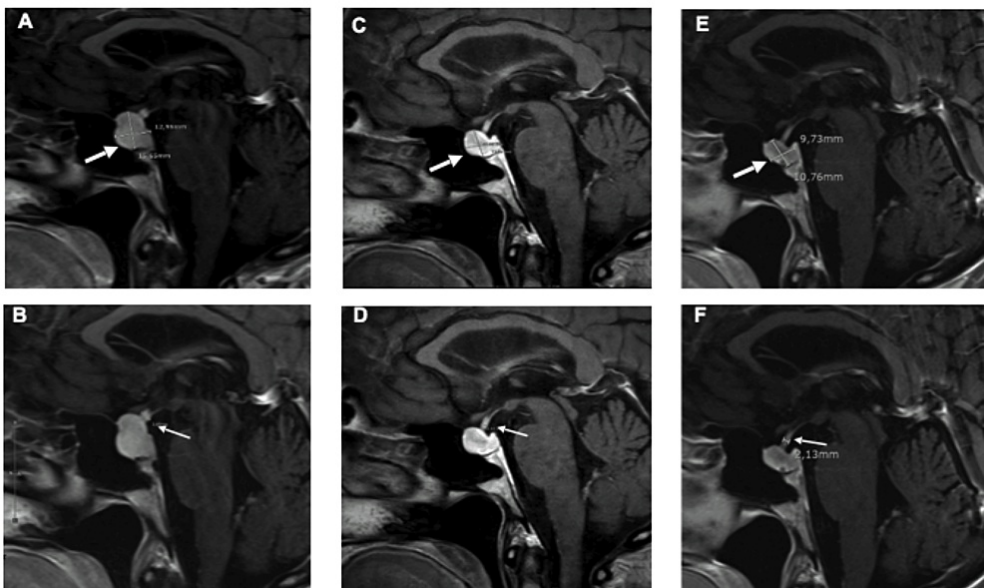
T1-weighted fluid-attenuated inversion recovery (FLAIR) sagittal MRI imaging features of the pituitary gland (thick arrows) and stalk (thin arrows) at diagnosis (A and B, respectively), three (C and D, respectively) and eight months after interruption of Pembrolizumab (E and F, respectively)

Gadolinium-enhanced magnetic resonance imaging (MRI) showed an enlarged pituitary gland (12.95 mm x 15.65 mm x 11.04 mm) with the homogeneous enhancement of the gland and the enlarged pituitary stalk (Figures 2A-2B). There was also a very discrete dural enhancement posterior of the sella turcica on the post-contrast images.

An increased hypothalamic signal was observed on fluid-attenuated inversion recovery (FLAIR) and T2-weighted images (not shown). Neurohypophysis depicted normal signals.

Differential diagnosis

In our case, a number of differential diagnoses were excluded. Among those, primary hypophysitis typically occurs in younger women during pregnancy or the peripartum period [4-5]. It tends to induce more enlargement of the pituitary gland than immunotherapy-induced hypophysitis (IIH). Pituitary size in IIH is typically less than two centimetres. Because of the greater size, patients with primary hypophysitis present more often with headaches and visual disturbances than patients with IIH [5]. This was not the case with our patient.

Pituitary lesions, such as adenoma, craniopharyngioma or Rathke’s cleft cyst, were ruled out in the imaging study. Moreover, the hypothalamic-pituitary-adrenal axis is usually affected last in such lesions [4].

Metastasis is another important differential diagnosis to rule out given the oncological context. The prevalence of isolated pituitary metastases is very low and accounts overall for only 0.4% of all intracranial metastases. Additionally, melanoma causes only 2% of all pituitary metastases [6]. In our case, there was a homogeneous enhancement of the pituitary gland, whereas heterogeneous enhancement is more typical for metastases [7].

Lastly, the clinical presentation of immunoglobulin G4 (IgG4)-related hypophysitis is very similar to IIH, but it was ruled out in our case based on the negative biochemical measurement of IgG4. Inconsistent with our case, the multisystemic disease is a more common presentation of IgG4-related disease. Isolated pituitary involvement is reported in only 4-5% of patients [4].

Management

The patient was initially treated with high-dose intravenous hydrocortisone substitution therapy, which was reduced to 25 mg hydrocortisone daily after seven days. Education on sick day rules and appropriate stress dosing was given. Thyroxine replacement with Levothyroxine 50 µg per day was initiated. According to international guidelines, checkpoint inhibition can be continued despite the development of an immune-related endocrinopathy, but this patient decided to interrupt treatment for personal reasons [8].

Outcome and follow-up

Symptoms drastically improved under replacement therapy. After three months of hormonal substitution and interruption of pembrolizumab, positron emission tomography of the head, thorax and abdomen as well as a new pituitary MRI showed no tumour recurrence or distant metastasis. Because of the stable disease, no alternative treatment was administered to optimise the control of the primary oncological disease (melanoma). MR images showed a reduced swelling of the pituitary gland and stalk after three months (Figures 2C-2D), and after eight months (Figures 2E-2F), no stigmata of residual inflammation could be observed. However, a small hypodense lesion between the anterior and posterior pituitary glands was reported. At the time of this report, one year after discontinuation of pembrolizumab, the patient remains clinically stable.

## Discussion

Immune checkpoint inhibitors are increasingly used in the treatment of various cancers. Therefore, immune-related adverse events are prevalent, including those affecting endocrine glands. Hypophysitis secondary to single-agent anti-PD-1-inhibitors, such as the most commonly used pembrolizumab and nivolumab, is very rare. The estimated incidence amounts to 0.5% of treated cases [2]. This is noticeably lower compared to that of hypophysitis due to anti-CTLA-4-inhibitors, which is estimated to be 10-15% [9-10]. Overall, little data is available on pembrolizumab-induced hypophysitis and consist mostly of case reports, series and letters.

**Figure 3:**
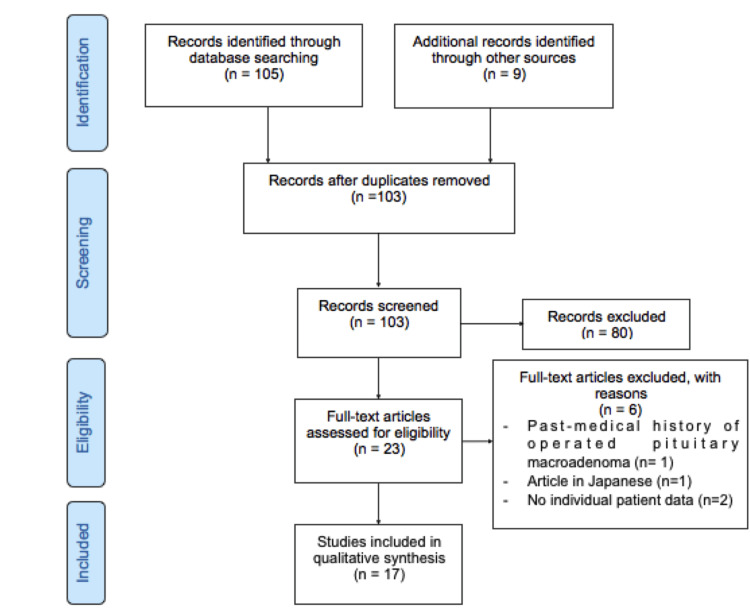
PRISMA flow chart summarizing studies identification, screening and selection PRISMA: Preferred Reporting Items for Systematic Reviews and Meta-Analyses

For comparison with our case, we conducted a systematic review of studies in MEDLINE from inception to 27/01/2022 using the search terms “hypophysitis”, “pituitary failure”, “pituitary insufficiency” and “pembrolizumab”. Data were selected from relevant studies according to variables described further in the study. The Preferred Reporting Items for Systematic Reviews and Meta-Analyses (PRISMA) flow diagram of study selection is depicted in Figure 3. We identified 17 papers reporting 20 patients with single-agent pembrolizumab-induced hypophysitis.

Clinical presentation

The time to onset after initiation of pembrolizumab therapy is about six months in most cases (range 1.5 to 39 months). Case reports by Boudjemaa et al. and Yamagata et al. suggest that auto-immune toxicity can develop even after treatment withdrawal, showing the importance of follow-up even after ending the treatment [11-12].

Patients with single-agent anti-PD-1 hypophysitis presented most frequently with general symptoms such as fatigue, anorexia, nausea and vomiting [13]. Patients were treated for melanoma (n=7; 33.3%), urogenital (n=5; 23.8%), lung (n=4; 19.0%), larynx (n=1; 4.8%), pharynx (n=1; 4.8%) and breast (n=1; 4.8%) and colon (n=1, 4.8%) neoplasms. This non-specific clinical presentation is often difficult to distinguish from the underlying oncological pathology in which such symptoms are common [14]. This could lead to delayed diagnosis [2]. However, concurrent hyponatremia could increase the clinical suspicion index of those symptoms as previously reported by others [9,15-19]. In our case, headaches but not visual disturbances were one of the presenting symptoms. Other potential etiologic factors of headache including metastasis were ruled out.

Hormonal disturbances

Anti-PD-1 hypophysitis is most commonly associated with isolated ACTH deficiency. This involves most frequently the hypothalamic-pituitary-adrenal axis. Defects of the neurohypophysis are mainly reported in the context of pituitary metastasis [4]. Ascertainment of secondary adrenal failure used different testing strategies, including morning cortisol and ACTH [9,11-12,15,17-27], short Synacthen test [12,16-18,21] and corticotropin-releasing hormone (CRH) stimulation test [12,16,24]. In our patient, three pituitary axes were affected: the adrenocorticotropic, thyroid and gonadotrophic axes. This is a rather rare presentation reported in three other patients in the currently available literature [20-22,27]. The time to onset was relatively short and ranged from three to nine months and two of the patients were diagnosed with melanoma [14]. Unlike our patient, the case reported by Malikova et al. presented another auto-immune adverse event involving the lungs (pneumonitis) [22]. However, based on the limited number of cases, one cannot accurately infer the risk of other organs’ involvement when multiple hormonal axes are impaired or the other way around. This seems not to be the case since independently of the number of hormonal axes involved, immune adverse events in other sites have been reported (Table 2) [9,11-12,15,17,19,21,25,28]. 

**Table 2:** Summary of included studies reporting cases with pembrolizumab-induced hypophysitis M: male, F: female, IIH: immunotherapy-induced hypophysitis, MRI: magnetic resonance imaging, irAE: immuno-related adverse events, ACTH: adrenocorticotrophic hormone, TSH: thyroid-stimulating hormone, fT4: free tetraiodothyronine, T3: triiodothyronine, SST: short Synacthen test, CRH: corticotropin-releasing hormone, LH: luteinizing hormone, FSH: follicular stimulating hormone, IGF-1: insulin-like growth factor 1, DHEAS: dehydroepiandrosterone sulfate, * years

Author, year	N°	Age*/Sex	Primary tumour	Time of IIH	Symptoms	Laboratory findings				Pituitary MRI	IrAEs other than ACTH deficiency
						Corticotropic axis	Thyrotropic axis	Other axis	Sodium		
Leiter, 2020 [20]	#1	75/M	Metastatic urothelial cancer	3.0 months	Severe fatigue, cold intolerance	Baseline ACTH 54 pg/mL, Random cortisol 21 µg/dL, Week 3: ACTH 13 pg/mL, Random cortisol 3 µg/dL	TSH 0.11 mIU/L, fT4 9.53 pmol/L, T3 2.69 pmol/L, Week 3: TSH 0.008 mIU/L, fT4 0.92 pmol/L	LH 2.21 IU/L, FSH 5.6 IU/L, Testosterone 76.42 ng/dL, Prolactin 2.9 ng/mL	Not available	Not available	Central hypothyroidism and hypogonadism
Doodnauth, 2021 [15]	#2	85/M	Metastatic urothelial cancer (high-grade urothelial pT1 cancer)	6.0 months	Generalized fatigue, appetite loss, abdominal pain, altered mental status	ACTH < 2 pg/mL, Cortisol 0.7 µg/dL	Month 14: TSH 35.86 mIU/L, Under substitution: TSH 0.82 mIU/L, fT4 6.31 pmol/L	LH 2.1 IU/L, FSH 3.0 IU/L, Testosterone 367 ng/dL, Prolactin 12.4 ng/ml, IGF-1 151	119 mmol/L	Normal	Primary thyroiditis
Hinata, 2021 [16]	#3	78/F	Metastatic ureteral cancer	5.5 months	Anorexia, general weakness, back pain, muscle pain in extremities, difficulties walking	ACTH 16.6 pg/ml, Cortisol 1.4 µg/dL, SST: Cortisol 0’: 1.4 µg/dL 30’: 6.2 µg/dL 60’: 8.5 µg/dL, CRH test: - Peak cortisol: 2.9 µg/dL - Peak ACTH: 16.7 pg/ml	TSH 2.86 mIU/L, fT4 18.80 pmol/L, T3 4.39 pmol/L	LH 23.2 IU/L, FSH 49.8 IU/L, Hyperkalemia and hyper chloremic metabolic acidosis, GH 0.15 µg/dL, Prolactin 20.8 ng/ml	134 mmol/L	Normal	None
Percik, 2019 [17]	#4	71/M	Transitional cell carcinoma	6.0 months	Fatigue, anorexia, diarrhoea, myalgia, depression	ACTH 13.1 pg/ml, Cortisol 1.16 µg/dL	TSH 1.99 mIU/L, fT4 10.1 pmol/L, T3 5.3 pmol/L	LH 7.3 IU/L, FSH 20.7 IU/L, Testosterone 126.90 ng/dL, Prolactin 34.3 ng/mL, GH 0.32 µg/dL, Renin (direct) 16.1 mIU/L, Aldosteron 274 pmol/L	< 135 mmol/L	Not specified	None
Boudjemaa, 2018 [11]	#5	60/M	Stage IV large cell lung carcinoma (cT2N2 M1b)	39.0 months (onset 15 months post-immunotherapy)	Fatigue, appetite loss, weight loss, nausea, pain in both shoulders	ACTH 0.506 pg/mL Cortisol: 0.1087 µg/dL	TSH < 0.05 mIU/L, fT4 15.28 pmol/L, T3 5.66 pmol/L	LH 5 IU/L, FSH 1.2 IU/L, Testosterone 775 ng/dl, Prolactin 22.5 ng/ml	Not available	Normal	Subclinical primary hyperthyroidism
Tanaka, 2020 [18]	#6	85/F	Stage IV squamous cell lung cancer (T3N2M1a)	6.0 months	Fatigue and loss of appetite	ACTH 8.3 pg/ml, Cortisol 0.92 µg/dL, SST: unsatisfactory cortisol response	TSH 3.39 mIU/mL, fT4 11.07 pmol/L, T3 4.07 pmol/L	LH 15.0 IU/L, FSH 50.9 IU/L, Estradiol 9.3 pg/ml, DHEAS 0.14 µmol/L, Prolactine 18.3 ng/mL, Progesterone < 0.05 ng/ml, GH 0.38 µg/dl, IGF-1 78 g/ml, ADH 3.1 pg/ml	122 mmol/L	Diffuse enhancement without enlargement	None
Chowdhury, 2020 [21]	#7	61/M	Stage IV lung adenocarcinoma	9.0 months	Weight loss, fatigue, breast pain while showering and minimal swelling of the breasts, no discharge	ACTH 9.536 pg/mL, Cortisol: <0.1087 µg/dL, SST: Cortisol 30’: 5.44 µg/dL 60’:11.24 µg/dL	TSH 0.2 mIU/L, fT4 8.42 pmol/L, T3 2.58 pmol/L, TPO neg TRAb neg	FSH 10.62 U/L, LH 6.90 U/L, Testosterone 467.24 ng/dL, SHBG 23 nmol/, Prolactin 24.85 ng/mL, IGF-1 91.79 ng/mL	135 mmol/L	Diffuse enlargement and heterogeneous enhancement	Secondary hypothyroidism, Minor skin rashes
Yamagata, 2019 [12]	#8	59/M	Relapsed adrenal metastatic non-small cell lung carcinoma (primary tumour: T2bN2M0, Stage IIIA)	7.5 months (Onset 4.0 months after Pembrolizumab discontinuation)	Anorexia, fatigue, fever	ACTH: 17.3 pg/mL, Cortisol: 0.89 µg/dL, CRH test: - Peak ACTH: 29.3 pg/ml - cortisol: 3.1 µg/dL, SST: Cortisol 0’: 2.3 µg/dL, 30’: not specified, 60’: 7.6 µg/dL, 24-h urinary cortisol : undetectable.	Baseline: Normal thyroid function anti-TPO Ab and anti-TgAb pos, Month 6: Primary hypothyroidism	LH 7.5 IU/L, FSH 33.2 IU/L, Testosterone 504 ng/dL, DHEAS: 0.46 µmol/L, Aldosterone 155.4 pmol/L, Renin activity 0.3 ng/mL/h, Prolactin 14.8 ng/mL, GH 0.052 µg/dL, IGF-1 99 g/mL	137 mmol/L	Normal	Primary hypothyroidism
Lupi, 2019 [9]	#9	80/M	Metastatic melanoma	10.5 months	Headache, severe muscle weakness	ACTH < 5 pg/mL, Cortisol 0.4 µg /dL	During L-thyroxine therapy: TSH 8 mIU/L, fT4 12.23 pmol/L, TgAb pos, TPOAb pos	LH: not specified, FSH 10 IU/L, Testosterone 220 ng/dL, Prolactine 19 ng/mL, IGF-1: 85 ng/mL	132 mmol/L	Normal	Primary hypothyroidism
Malikova, 2018 [22]	#10	65/F	Metastatic melanoma (primary tumour: T4aN2a M0, stage IIIc)	3.0 months	Headache, fever, fatigue, cough, anorexia	ACTH 4.45 pg/mL, Cortisol 1.34 µg/dL	TSH 0.049 mIU/L, fT4: not specified	LH 0.9 IU/L, FSH 19.5 IU/L	Not available	Peripheral enhancement with discreet non-homogeneity	Pneumonitis, Secondary hypothyroidism and hypogonadism
Wei, 2019 [29]	#11	24/F	Metastatic melanoma	25.5 months	Nausea, vomiting	Corticotroph defect without further specification	Not specified	Not specified	Not available	Not available	Primary hypothyroidism
Percik, 2019 [17]	#12	65/M	Melanoma	16.0 months	Fatigue, anorexia, weight loss	ACTH 11.8 pg/mL, Cortisol 4.28 µg/dL, SST: Cortisol 0’: 2.16 µg/dL, 30’: 6.74 µg/dL, 60’: 9.10 µg/dL	TSH 3.46 mIU/L, fT4 9 pmol/L, T3 5.1 pmol/L	LH 5.7 IU/L, FSH 9.9 IU/L, Testosterone 216.32 ng/dL, DHEAS 0.5 µmol/L, Prolactine < 0.5 ng/mL, IGF-1 195.05 ng/mL, Renin direct 6 mIU/L, Aldosterone 249 pmol/L	Normal	Not specified	None
Do, 2021 [23]	#13	53/F	Metastatic melanoma (cTx, pN1b, M1)	9.0 months	Progressive generalized weakness, extreme fatigue, lethargy, myalgia, poor appetite, weight loss, mood changes	ACTH < 1.1 pg/mL Cortisol 0.2 µg/dL	Not specified	LH normal, FSH normal, Prolactin normal	Not available	Not available	None
Current case	#14	55/F	Melanoma (pT3aN1a)	3.0 months	Headache, nausea and fatigue	ACTH<0.5 pg/mL, Cortisol 0.5 µg/dL	TSH 0.24 mIU/L, fT4 9.5 pmol/L, T3 3.25 pmol/L	LH 0.2 IU/L, FSH 0.2 IU/L, Oestradiol < 11.0 ng/L, IGF-1 88.6 ng/mL	138 mmol/L	Diffuse enlargement with homogeneous enhancement	Secondary hypothyroidism and hypogonadism
Montero Pérez O, 2022 [27]	#15	79/M	Melanoma	6.0 months	Dysphagia, early fullness, nausea, vomiting, diarrhoea, asthenia and weight loos	ACTH 2 pg/mL, Cortisol 7.6 µg/dL	TSH 0.061 mIU/L, fT4 11.45 pmol/L	LH normal, FSH normal, Testosterone 3 ng/dL	130 mmol/L	Normal	Secondary hypothyroidism and hypogonadism
Yamamoto, 2021 [24]	#16	78/M	Metastatic hypo-pharyngeal cancer	7.5 months	Fever, anorexia, vomiting	Cortisol: 0.6 µg/dL, 24h urinary cortisol: undetectable, CRH test: no response of ACTH or cortisol	Not specified	Anterior pituitary hormones and loading tests: normal. ADH 3.0 pg/mL	135 mmol/L	Normal	None
Percik, 2019 [17]	#17	51/F	Breast carcinoma	6.0 months	Fatigue, diarrhoea, myalgia	ACTH < 5 pg/mL, Cortisol < 1.0 µg/dL, SST: Cortisol 0’: < 27.6 µg/dL, 30’: 28.7 µg/dL, 60’: 42.8 µg/dL	TSH 2.81 mIU/L, fT4 7.8 pmol/L, T3 5.7 pmol/L	DHEAS < 0.41 µmol/L	Normal	Not specified	Pneumonitis
Oristrell, 2018 [19]	#18	55/F	Infiltrating ductal breast carcinoma (cT2cN1c M0)	12.0 months	Pericardial chest pain, hypotension	ACTH <1.6 pg/mL, Cortisol 0.93 µg/dL	TSH 16.819 mIU/L, fT4 1.02 pmol/L	Not specified	132 mmol/L	Normal	Pericarditis with Cardiac tamponade, Pancytopenia
Percik, 2019 [17]	#19	58/F	Ovary carcinoma	4.0 months	Fatigue, anorexia	ACTH 10.8 pg/mL, Cortisol 3.27 µg/dL	TSH 1.74 mIU/L, fT4 10.5 pmol/L, T3 5.3 pmol/L	LH 31.8 IU/L, FSH 76.7 IU/L, Prolactine 13.9 ng/mL, IGF -1 74.96 ng/mL	< 135 mmol/L	Not specified	None
Oguz, 2021 [25]	#20	49/M	Stage III laryngeal carcinoma (T3N1M0)	7.5 months	Weakness, appetite loss, weight loss, nausea and vomiting	ACTH 10.1 pg/mL, Cortisol: 0.47 µg/dL	TSH 4.46 mIU/mL, fT4 11.1 pmol/L, T3 8.76 pmol/L	Prolactin 46.1 ng/ml, Other anterior pituitary hormones: normal, No signs of diabetes insipidus, LH not specified, FSH not specified, Testosterone not specified, DHEAS 0.62 µmol/L	Normal	Mild enlargement with heterogeneous enhancement	Transient primary hypothyroidism, Possible hepatitis and pancreatitis
Bekki, 2020 [26]	#21	65/F	Metastatic colon cancer (primary tumour: stage III)	3.0 weeks	Fatigue	ACTH 3.0 pg/mL, Cortisol 0.5 µg/dL	Not specified.	Other anterior pituitary hormones: normal	Not available	Normal	Not available

Imaging findings in pembrolizumab-associated hypophysitis

In the absence of a biopsy to ascertain the diagnosis of hypophysitis, imaging studies are critical tools in daily clinical practice. In our case, we describe diffuse enlargement and homogeneous enhancement of the gland as well as moderate enlargement with the enhancement of the pituitary stalk found on magnetic resonance imaging. This is however not a widespread finding since some reported a solitary change in homogeneity or enhancement [18,22] and others normal imaging findings [9,11-12,15,17,19,27]. As previously reported [29], the review of published cases and our case suggest that increased pituitary size is associated with multiple hormonal axis involvement [21,25,27]. As opposed to the enlargement and enhancement of the pituitary stalk, the same changes on the gland seem to be more specific for the diagnosis of hypophysitis using MR imaging and therefore are more accurate for follow-up purposes [30].

Comparison with anti-CTLA-4-associated hypophysitis

Anti-PD-1 hypophysitis, specifically that associated with pembrolizumab, seems to be a different clinical entity than anti-CTLA-4 hypophysitis [14]. According to Faje et al., when treated with pembrolizumab (or nivolumab), patients typically tend to develop hypophysitis later during the course of treatment compared to their ipilimumab-treated counterparts (median 25.8 weeks vs. 9.3 weeks, p < 0.0001) [2].

Another difference between anti-PD-1 therapy and anti-CTLA-4 therapy is the presence of MRI changes. Patients on anti-CTLA-4 therapy usually have an enlargement of the pituitary gland on MRI imaging, which often resolves within a few months [5]. Sometimes, MRI changes may even precede the clinical picture, which we observed in our case. It is recommended to perform MRI imaging at baseline and routinely in the first six months after initiation of immunotherapy. An incidental finding of pituitary enlargement should lead to a biochemical assessment of all pituitary axes. However, pituitary enlargement or other MRI changes are barely seen after anti-PD-1 monotherapy [2].

On the other hand, similarly to anti-CTLA-4-induced endocrine adverse events [31], except for one patient in whom de-escalation of corticosteroids and thyroid hormone supplementations were considered [27]. All patients with available data required hormonal support after a follow-up time ranging from one to 19 months. In most cases, pembrolizumab was continued [9,17,25]. In some instances, this was after a transient interruption [20,23].

Strengths and limitations

The strengths of the present report include a succinct case description and a systematic review of the literature. Comparison with similar published reports enabled us to identify a common presenting feature that could be characteristic of single-agent pembrolizumab-associated hypophysitis with the involvement of multiple hormonal axes. The absence of pituitary biopsy to ascertain the diagnosis of immune-mediated hypophysitis could be considered a limitation of our study. Nevertheless, the benefit-risk ratio of this diagnostic modality needs to be considered due to its invasive nature [4]. To date, there are no clear recommendations nor clear criteria for considering pituitary biopsy in adults. A biopsy could be requested when the diagnosis is unclear [4]. In our case, clinical and biological evaluations enabled us to rule out potential differentials.

## Conclusions

We report a case of single-agent pembrolizumab-induced hypophysitis characterized by early disease onset after anti-PD-1 treatment initiation, panhypopituitarism and increased pituitary mass. These are distinct features compared to the majority of reported cases of single-agent pembrolizumab-induced hypophysitis, as most patients present with an isolated ACTH deficiency. Further exploration to ascertain whether or not this is a new clinical entity warrants further investigation. Until then, clinicians should be aware that hypophysitis induced by single-agent pembrolizumab might cover a heterogeneous clinical spectrum. Prompt identification and treatment remain of great importance to prevent further deterioration.
