# Correction: Exploring a New Entity of Single-Agent Pembrolizumab-Associated Hypophysitis

**DOI:** 10.7759/cureus.c166

**Published:** 2024-03-20

**Authors:** Eric Balti, Sarah Verhaeghe, Vibeke Kruse, Stijn Roels, Peter Coremans

**Affiliations:** 1 Department of Endocrinology and Diabetes, VITAZ Hospital, Sint-Niklaas, BEL; 2 Department of Medical Oncology, VITAZ Hospital, Sint-Niklaas, BEL; 3 Department of Molecular Imaging, Pathology, Radiotherapy and Oncology (MIPRO), University of Antwerp, Wilrijk, BEL; 4 Department of Oncology, Antwerp University Hospital, Edegem, BEL; 5 Department of Radiology, VITAZ Hospital, Sint-Niklaas, BEL

This article has been corrected to accurately reflect author Vibeke Kruse’s affiliations. The following affiliations have been added:

Department of Molecular Imaging, Pathology, Radiotherapy and Oncology (MIPRO), University of Antwerp, Wilrijk, Belgium Department of Oncology, Antwerp University Hospital, Edegem, Belgium

The authors deeply regret that this was not noticed prior to publication.

 

